# Impact of preoperative smoking on patients undergoing right hemicolectomies for colon cancer

**DOI:** 10.1007/s00423-022-02486-9

**Published:** 2022-03-15

**Authors:** Sarit Badiani, Jason Diab, Evangeline Woodford, Pragadesh Natarajan, Christophe R. Berney

**Affiliations:** 1 Bankstown Lidcombe Hospital, Eldridge Rd, Bankstown, NSW 2200 Australia; 2 School of Medicine, University of New South Wales, Sydney, Australia; 3 School of Medicine, University of Notre Dame, Sydney, Australia; 4 Department of Surgery, Westmead Hospital, Westmead, NSW Australia

**Keywords:** Colorectal cancer, Smoking, Outcomes, Complications

## Abstract

**Purpose:**

The tobacco epidemic is one of the biggest global public health issues impacting quality of life and surgical outcomes. Although 30% of colon cancers warrant a right hemicolectomy (RH), there is no specific data on the influence of smoking on postoperative complications following RH for cancer. The aim of this study was to determine its effect on post-surgical outcomes.

**Methods:**

Patients who underwent elective RH for colon cancer between 2016 and 2019 were identified from the ACS-NSQIP database. Propensity score matching (PSM) was used with a maximum absolute difference of 0.05 between propensity scores. Primary outcome was to assess the 30-day complication risk profile between smokers and non-smokers. Secondary outcomes included smoking impact on wound and major medico-surgical complication rates, as well as risk of anastomotic leak (AL) using multivariable logistic regression models.

**Results:**

Following PSM, 5652 patients underwent RH for colon cancer with 1,884 (33.3%) identified as smokers. Smokers demonstrated a higher rate of organ space infection (4.1% vs 3.1%, *p* = 0.034), unplanned return to theatre (4.8% vs 3.7%, *p* = 0.045) and risk of AL (3.5% vs 2.1%, *p* = 0.005). Smoking was found to be an independent risk factor for wound complications (OR 1.32, 95% CI 1.03–1.71, *p* = 0.032), primary pulmonary complications (OR 1.50, 95% CI 1.06–2.13, *p* = 0.024) and AL (OR 1.66, 95% CI 1.19–2.31, *p* = 0.003).

**Conclusion:**

Smokers have increased risk of developing major post-operative complications compared to non-smokers. Clinicians and surgeons must inform smokers of these surgical risks and potential benefit of smoking cessation prior to undergoing major colonic resection.

## Introduction

Colorectal cancer (CRC) is the fourth most common malignancy and the second most common cause of cancer death globally [1]. Right-sided colon cancer accounts for approximately 30% of all large bowel cancers and commonly treated by right hemicolectomy (RH) [2]. The tobacco epidemic is one of the biggest public health issues and accounts for the death of approximately 8 million people worldwide [3]. Latest prevalence estimates that 49% of men and 8% of women smoke in low- and middle-income countries with a ratio that varies slightly in higher income nations (37% for men and 21% for women). Despite active public health interventions such as negative advertising, tax increase and age restrictions, smoking remains a significant global problem with the highest percentage of smokers aged between 45 and 64 years old [4]. With high prevalence of smokers and increasing rates of colon cancer, clinicians and surgeons are more likely to encounter patients with newly diagnosed right colon cancer who are long-term smokers.

Most surgical literature strongly supports the negative association of long-term preoperative smoking and increased complications rates in both gastrointestinal (GI) and non-GI surgery [5–13]. These studies are consistent in demonstrating higher rates of post-operative morbidity such as wound infections, pulmonary complications and increased anastomotic leaks (ALs). Consequently, this translates to a substantial financial cost to the health system [14–16]. However, many studies assessing the impact of preoperative smoking on GI surgery are quite heterogenous consisting of a variety of operation types and pathologies, therein reducing its overall specificity [6–8]. To our knowledge, there is no data assessing the direct impact of preoperative smoking on RH.

The aim of this study was to use the American College of Surgeons National Surgical Quality Improvement Project (ACS-NSQIP) database to quantify the complication risk profile in smokers undergoing a RH for colon cancer, compared with non-smokers. This would support clinicians and surgeons to objectively evaluate what the negative influence of smoking is on post major colonic resection and inform patients of their associated risks compared to non-smokers.

## Methods

### Study population

The ACS-NSQIP is a national validated database which prospectively collects global data from more than 600 hospitals worldwide (including our own institution) with over 200 targeted validated outcome variables. Sampling strategy, data abstraction and outcomes recorded using this database have been previously validated [17, 18]. This database was queried for patients with a primary postoperative diagnosis of right sided colon cancer between 2016 and 2019 based on the International Classification of Disease, Ninth (ICD-9) and Tenth (ICD-10) revision codes. Only those with pathologic confirmation of malignant neoplasm of the caecum, ascending colon, hepatic flexure and proximal transverse colon were included. The primary diagnosis was then cross-referenced with the Current Procedural Terminology (CPT) codes 44204 (laparoscopy colectomy partial w/anastomosis), 44205 (laparoscopy, surgical; colectomy, partial, with removal of terminal ileum with ileocolostomy), 44140 (colectomy partial w/anastomosis) and 44160 (colectomy, partial, with removal of terminal ileum with ileocolostomy). Patients who underwent either a laparoscopic or robotic procedure were coded as a minimally invasive procedure (MIP). If patients were coded to having a MIP with an unplanned conversion to open, then this was coded as a “MIP converted to open.” Open procedures were classified as a separate group. Due to the use of de-identified patient data only, this study is considered non-human subjects research and no ethics is required.

### Inclusion and exclusion criteria

To allow for a more homogenous and meaningful analysis of data, we excluded all patients who underwent emergency RH, as well as those with a preoperative American Society of Anesthesiologists (ASA) class of 5 (moribund), systemic inflammatory response syndrome (SIRS), sepsis or septic shock, acute renal failure, ventilator dependence and pneumonia.

### Predictor variables

Patients were grouped into smokers and non-smokers defined as smoking cigarettes within 12 months prior to surgery, as defined by the NSQIP database. Standard reported patient demographics as provided by the NSQIP database includes age, sex, race and ethnicity. Patient clinical characteristics included body mass index (BMI), ASA classification, preoperative weight loss > 10% of total weight, functional status and chronic steroid use. Preoperative medical comorbidity variables included insulin-dependent (IDDM) or non-insulin-dependent diabetes mellitus (NIDDM), dyspnoea, chronic obstructive pulmonary disease (COPD), hypertension and disseminated cancer. Surgical characteristics of interest included operative time, procedure type (open, laparoscopic, robotic) and wound classification.

### Outcomes

Outcomes evaluated included complications that occurred within 30 days of surgery as validated by the NSQIP database: SSI (including superficial, deep or organ space types), wound dehiscence, sepsis, Clostridium difficile (C. diff) infection, urinary tract infection (UTI), pneumonia, reintubation, deep vein thrombosis (DVT), pulmonary embolism (PE), stroke, myocardial infarction (MI), renal failure or injury, bleeding requiring > 4 unit transfusion, return to the operating room, prolonged length of stay (LOS) defined by patients in 4th quartile for LOS (8 days), hospital readmission, anastomotic leak (AL), prolonged NPO/nasogastric tube > 48 h (ileus), or all-cause mortality.

Composite outcomes of interest evaluated included:

1. Wound complications (superficial and/or deep SSI, and wound dehiscence)
2. Surgical complications (wound complications, organ space infection, unplanned return to operating room, bleeding requiring transfusion and AL)
3. Medical complications (pneumonia, PE, failure to wean from ventilator and reintubation, progressive or acute renal insufficiency (creatinine > 2 mg/dL), MI, cardiac arrest with or without cardiopulmonary resuscitation)
4. Primary pulmonary complications (pneumonia, reintubation and failure to wean from ventilator)
5. Embolic complications (DVT/PE)
6. Renal complications [acute or progressive renal failure (creatinine > 2 mg/dL)]
7. Septic complications (sepsis or septic shock). Sepsis was evaluated separately as its aetiology could have been either medical or surgical without any clear distinction from the NSQIP database.

### Statistical analysis

Propensity score matching was performed using SAS 9.4 (SAS Institute Inc., Cary, NC). Propensity scores were calculated based on the patient’s clinical characteristics and preoperative medical comorbidities as previously listed (age, sex, race, BMI, ASA classification, preoperative weight loss > 10% of total weight, functional status, chronic steroid use, diabetes mellitus, dyspnoea, chronic obstructive pulmonary disease (COPD), hypertension and disseminated cancer). 2:1 matching was used with a maximum absolute difference of 0.05 between propensity scores, with matching optimized for number of matches. Normally distributed continuous variables were analysed using two-sample Student’s *t* tests, whilst categorical variables were assessed with chi-square testing. Odds ratios (OR) of the composite and individual outcomes of interest were calculated using both univariate logistic analysis and multivariate regression (age, sex, functional status, diabetes, COPD, congestive heart failure, medicated hypertension, chronic steroid use, BMI and ASA class were considered within the model)*.* Observations with missing values were censored from analyses.

## Results

### Demographics

The original population had 17,033 patients who underwent elective RH for colon cancer with 1,884 (11.1%) who identified as smokers (Table 1). There was an almost equal distribution of male to female smokers (51.3% vs 48.7%, *p* = 0.005). Smokers were significantly younger than non-smokers (64 years vs 70 years, *p* < 0.001). The mean operation time was 155 min (*SD* = 70 min) with smokers yielding a significantly longer operative time of 4 min (158 vs 154, *p* = 0.005). Smokers also had a significantly longer LOS of 0.25 days compared to non-smokers (5.2 days vs 4.95 days, *p* = 0.001). The most common ethnicity group was Caucasians (68.4%), followed by African Americans (9.6%) and Asians (3.6%). There were 3,650 (21.4%) patients with diabetes mellitus of which 2,495 (14.6%) were NIDDM and 1,155 (6.8%) IDDM. The mean BMI was 28.8 kg/m^2^ (*SD* = 6.9), with smokers having a significantly lower BMI distribution compared to non-smokers (*p* < 0.001). Most patients (60.1%) had a preoperative ASA score of 3, though smokers were significantly more likely to have a higher ASA grade (*p* < 0.001). Most procedures were performed as a MIP in 13,187 cases (77.4%), followed by planned open surgery 2,743 (16.1%), 1,103 (6.5%) started as a MIP then converted to open.

**Table 1:** Patient demographics and operative characteristics by smoking status: original and propensity score-matched population. *COPD* chronic obstructive pulmonary disorder, *BMI* body mass index, *CHF* chronic heart failure, *ASA* American Society of Anaesthesiologists, *MIP* minimally invasive procedure

Variables	Original population				Propensity score-matched population			
	Non-smoker (%)	Smoker (%)	Total (%)	*p* value	Non-smoker (%)	Smoker (%)	Total (%)	*p* value
Number	15,149 (88.9)	1884 (11.1)	17,033 (100)	**N/A**	3768 (66.7)	1884 (33.3)	5652 (100.0)	**N/A**
Age	70 (± 12)	64 (± 11)	70 (± 12)	** < 0.001**	66 (± 13)	64 (± 11)	65 (± 12)	** < 0.001**
Operative time	154(± 69)	158 (± 74)	155 (± 70)	**0.005**	157 (± 74)	158 (± 74)	158 (± 74)	0.734
Sex (male)	6901 (45.6)	966 (51.3)	7867 (46.2)	** < 0.001**	1848 (49.0)	966 (51.3)	2838 (50.2)	0.114
Ethnicity								
American Indian/Alaska Native	61 (0.4)	10 (0.5)	71 (0.4)	** < 0.001**	19 (0.5)	10 (0.5)	29 (0.5)	0.573
Asian	576 (3.8)	34 (1.8)	610 (3.6)		87 (2.3)	34 (1.8)	121 (2.1)	
Black/African American	1374 (9.1)	261 (13.9)	1635 (9.6)		472 (12.5)	261 (13.9)	733 (13.0)	
Native Hawaiian/Pacific Islander	28 (0.2)	79 (0.4)	35 (0.2)		10 (0.3)	7 (0.4)	17 (0.3)	
Unknown	2688 (17.7)	342 (18.2)	3030 (17.8)		687 (18.2)	342 (18.2)	1029 (18.2)	
White	10,422 (68.8)	1230 (65.3)	11,652 (68.4)		2493 (66.2)	1230 (65.3)	3723 (65.9)	
BMI								
Underweight	139 (0.9)	58 (3.1)	197 (1.2)	** < 0.001**	50 (1.4)	52 (2.8)	102 (1.9)	**0.008**
Normal	4189 (27.7)	642 (34.8)	4831 (28.4)		1214 (33.2)	642 (34.6)	1856 (33.7)	
Overweight	5186 (34.2)	555 (30.1)	5741 (33.7)		1098 (30.0)	555 (29.9)	1653 (30.0)	
Obese class 1	3143 (20.7)	335 (18.3)	3478 (20.4)		719 (19.7)	335 (18.1)	1054 (19.1)	
Obese class 2	1404 (9.3)	168 (9.1)	1572 (9.2)		351 (9.6)	168 (9.1)	519 (9.4)	
Obese class 3	978 (6.5)	97 (5.3)	1075 (6.3)		212 (5.8)	97 (5.2)	309 (5.6)	
Preoperative comorbidity								
Diabetes	3320 (21.9)	330 (17.5)	3650 (21.4)	** < 0.001**	713 (18.9)	330 (17.5)	1043 (18.5)	0.199
COPD	772 (5.1)	270 (14.3)	1042 (6.1)	** < 0.001**	399 (10.6)	270 (14.3)	669 (11.8)	** < 0.001**
CHF	154 (1.0)	21 (1.1)	175 (1.0)	0.691	36 (1.0)	21 (1.1)	57 (1.0)	0.572
Disseminated cancer	1030 (6.8)	168 (8.9)	1198 (7.0)	** < 0.001**	313 (8.3)	168 (8.9)	481 (8.5)	0.438
Steroid use	534 (3.5)	57 (3.0)	591 (3.5)	0.264	132 (3.5)	57 (3.0)	189 (3.3)	0.346
Hypertensive med	9038 (59.7)	966 (51.3)	10,004 (58.7)	** < 0.001**	2001 (53.1)	966 (51.3)	2967 (52.5)	0.194
ASA class								
ASA 1	160 (1.1)	10 (0.5)	170 (1.0)	** < 0.001**	20 (0.5)	10 (0.5)	30 (0.5)	0.367
ASA 2	4803 (31.7)	526 (27.9)	5329 (31.3)		1137 (30.2)	526 (27.9)	1663 (29.4)	
ASA 3	9146 (60.4)	1214 (64.4)	10,360 (60.8)		2344 (62.2)	1214 (64.4)	3558 (63.0)	
ASA 4	1040 (6.9)	134 (7.1)	1174 (6.9)		267 (7.1)	134 (7.1)	401 (7.1)	
Procedure type								
MIP	11,852 (78.3)	1335 (70.9)	13,187 (77.4)	** < 0.001**	2898 (76.9)	1335 (70.9)	4233 (74.9)	** < 0.001**
MIP converted to open	958 (6.3)	145 (7.7)	1103 (6.5)		249 (6.6)	145 (7.7)	394 (7.0)	
Open	2339 (15.4)	404 (21.4)	2743 (16.1)		621 (16.4)	404 (21.4)	1025 (18.1)	

Bold face text indicates a significant statistical value

Following propensity score matching, 5,652 patients who underwent elective RH for colon cancer were selected with 1,884 (33.3%) who identified as smokers (Table 1). Smokers remained significantly younger than non-smokers (64 years vs 66 years, *p* < 0.001). There was no significant difference between the mean operation time for smokers and non-smokers (*p* = 0.734), with an overall mean of 158 min (*SD* = 74 min). In relation to preoperative comorbidities, there were 1,043 (18.5%) patients with diabetes mellitus; of these, 726 (12.9%) were NIDDM and 317 (5.6%) IDDM, with no significant difference between smokers and non-smokers (*p* = 0.199). COPD remained more significant in the smokers than non-smokers (14.3% vs 10.6, *p* < 0.001). The mean BMI was 28.8 kg/m^2^ (*SD* = 6.9). Most patients (63.0%) had a preoperative ASA score of 3 with no difference in ASA distribution between smokers and non-smokers (*p* = 0.367). Most procedures were performed as a MIP in 4,233 cases (74.9%), followed by planned open surgery 1,025 (18.1%), and 394 (7.0%) started as a MIP then converted to open.

### Outcomes

Analysis of 30-day outcomes of individual complications is summarised in Table 2. Of clinical significance, smokers were found to have a higher rate of organ space infection (4.1% vs 3.1%, *p* = 0.034), acute renal failure (0.6% vs 0.1%, *p* = 0.001), unplanned return to the operating room (4.8% vs 3.7%, *p* = 0.045) and anastomotic leak (3.5% vs 2.1%, *p* = 0.005). The rate of AL was observed in 144/5652 (2.5%) patients. Higher proportion of wound-related complications (superficial SSI, deep SSI and wound dehiscence) was observed in smokers but of no clinical significance. Similarly, also not significant, there was a trend towards higher risk of pneumonia, reintubation and sepsis in smokers.

**Table 2:** Propensity score-matched population—incidence of individual complications by smoking status. *SSSI* superficial surgical site infection, *DSSI* deep surgical site infection, *CVA* cerebrovascular accident, *DVT* deep venous thrombosis, *CPR* cardiopulmonary resuscitation

30-day outcome	Non-smoker (*n* = 3768)	Smoker (*n* = 1884)	Total (*n* = 5652)	*p* value
SSSI	72 (1.9)	43 (2.3)	115 (2.0)	0.351
DSSI	12 (0.3)	12 (0.6)	24 (0.4)	0.083
Dehiscence	22 (0.6)	17 (0.9)	39 (0.7)	0.173
Organ space infection	115 (3.1)	78 (4.1)	193 (3.4)	0.034*
Pneumonia	58 (1.5)	41 (2.2)	99 (1.8)	0.085
Reintubation	39 (1.0)	29 (1.5)	68 (1.2)	0.101
Pulmonary embolism	17 (0.5)	10 (0.5)	27 (0.5)	0.682
On Ventilator (> 48 h)	29 (0.8)	16 (0.9)	45 (0.8)	0.751
Progressive renal insufficiency	17 (0.5)	8 (0.4)	25 (0.4)	0.887
Acute renal failure	5 (0.1)	12 (0.6)	17 (0.3)	0.001*
Urinary tract infection	53 (1.4)	20 (1.1)	73 (1.3)	0.279
Stroke/CVA	6 (0.2)	5 (0.3)	11 (0.2)	0.523
Cardiac arrest requiring CPR	14 (0.4)	8 (0.4)	22 (0.4)	0.763
Myocardial infarction	23 (0.6)	10 (0.5)	33 (0.6)	0.711
Transfusions/Intraop/Postop	289 (7.7)	128 (6.8)	417 (7.4)	0.235
DVT requiring therapy	30 (0.8)	20 (1.1)	50 (0.9)	0.315
Sepsis	69 (1.8)	43 (2.3)	112 (2.0)	0.251
Septic shock	47 (1.3)	24 (1.3)	71 (1.3)	0.933
Return to operating room	138 (3.7)	90 (4.8)	228 (4.0)	0.045*
Readmission	347 (9.2)	173 (9.2)	520 (9.2)	0.974
C difficile	45 (1.2)	23 (1.2)	68 (1.2)	0.931
Anastomotic leak	78 (2.1)	66 (3.5)	144 (2.6)	0.005*
30-day mortality	26 (0.7)	11 (0.6)	37 (0.7)	0.831

Bold face text indicates a significant statistical value

After adjusting for patient comorbidities and operative approach, multivariable analysis for selected outcomes and composite outcomes demonstrated smoking to be an independent risk factor for wound complications (OR 1.32, 95% CI 1.03–1.71, *p* = 0.032), primary pulmonary complications (OR 1.50, 95% 1.06–2.13, *p* = 0.024) and AL (OR 1.66, 95% CI 1.19–2.31, *p* = 0.003). Major medical complications (OR 1.15, 95% CI 0.97–1.36, *p* = 0.114), surgical complications (OR 1.09, 95% CI 0.93–1.29, *p* = 0.282), embolic complications (OR 1.27, 95% CI 0.77–2.07, *p* = 0.797), renal (OR 1.72, 95% CI 0.92–3.22, *p* = 0.091) and sepsis (OR 1.19, 95% CI 0.87–1.63, *p* = 0.284) trended towards a higher risk for smokers but did not reach statistical significance (Table 3).

**Table 3:** Propensity score-matched population—odds ratios of complications, both unadjusted and adjusted for age, sex, functional status, BMI, history of diabetes mellitus, COPD, congestive heart failure, medicated hypertension, chronic steroid use and American Society of Anesthesiologists classification. All endpoints represent 30-day outcomes. Significant outcomes bold highlighted

	Unadjusted OR	95% CI	***p*** value	Adjusted OR	95% CI	***p*** value
Wound complications	1.30	1.01–1.66	**0.041**	1.32	1.03–1.71	**0.032**
Major medical complications	1.14	0.97–1.34	0.122	1.15	0.97–1.36	0.114
Primary pulmonary complication	1.41	1.01–1.96	**0.042**	1.50	1.06–2.13	**0.024**
Surgical complications	1.11	0.95–1.30	0.200	1.09	0.93–1.29	0.282
Return to operating room	1.32	1.01–1.73	**0.045**	1.32	1.00–1.74	0.052
Renal	1.83	1.00–3.36	0.052	1.72	0.92–3.22	0.091
Sepsis or septic shock	1.14	0.84–1.55	0.394	1.19	0.87–1.63	0.284
Embolic	1.31	0.81–2.11	0.274	1.27	0.77–2.07	0.797
Anastomotic leak	1.69	1.22–2.33	**0.002**	1.66	1.19–2.31	**0.003**

## Discussion

Clinicians and surgeons will encounter patients with a newly diagnosed colon cancer who are long-term smokers, often at a relatively young age. Indeed, we identified that smokers were significantly younger than non-smokers by an average of 6 years (*p* < 0.001) in the original population. This can be explained by the fact that smokers have an estimated higher risk of developing colorectal cancer of 20 to 60% [19]. Studies that have evaluated the complication risk profile of chronic smokers undergoing GI surgery have reported an increased rate of impaired wound healing, increased infection and cardiopulmonary complication rates [6, 7, 9]. This has a detrimental impact on patients’ wellbeing and quality of life. In addition, this also poses a financial burden on health care resources. It is therefore beneficial to risk stratify long-term smokers undergoing surgery and mitigate those risks in the preoperative period. Our analysis of the NSQIP database on elective RH for bowel cancer remains consistent with current literature demonstrating increased wound and pulmonary complications, as well as AL rates amongst smokers compared with non-smokers. The challenge therein lies in the effective perioperative management in this group of patients, including national pre-operative smoking cessation programmes that could potentially mitigate their complication risk.

Using the NSQIP database, Sharma and colleagues analysed over 47,000 patients who underwent a colonic resection for benign (diverticular disease and inflammatory bowel disease) and malignant (colorectal cancer) pathologies, demonstrating almost a 1.5-fold increase in morbidity and mortality amongst smokers [6]. Similarly, Brajcich and colleagues reported higher rates of death and serious postoperative morbidity amongst chronic smokers in patients undergoing various GI procedures (colorectal, pancreatic, gastric or hepatic procedures) [7]. Both studies included large sample size, but the heterogeneity of selected patients potentially masks the real impact of smoking on specific operation types, thus limiting the true understanding of its explicit procedure-related risk. In comparison, our study offers a more homogenous picture, therefore reducing variability and heterogeneity, whilst adding clearer risk stratification for patients undergoing RH.

Wound complications, in particular SSIs, are the third most reported type of healthcare-associated infections. Their management is labour intensive and is associated with prolonged LOS and an additional economic burden [20]. Long-term smoking increases the likelihood of SSIs and wound dehiscence. A large systematic review and meta-analysis assessing wound complications in smokers across a range of surgical specialties reported a twofold increase in adjusted OR for healing delay and wound dehiscence, 1.8-fold increase in SSI and 2.3-fold increase in overall wound complication rates [21]. Brajcich et al. and Sharma et al. similarly reported approximately a 1.3-fold increase in wound complications of clinical significance [6, 7]. In our study, smokers also had a 1.3-fold increased risk of wound complications, confirming its negative impact that is in line with current literature. Wound complications are also influenced by the use of long-term steroids [22]. Ismael et al. analysed over 600,000 patients from the NSQIP database and reported in those patients with steroid use, increased SSI rates from 2.9 to 5.0% (*OR* = 1.724), increased deep SSIs from 0.8 to 1.8% (*OR* = 2.353), and wound dehiscence increased risk of 2- to threefold (*OR* = 3.338). In our study, 3.0% of smokers were on long-term steroids compared with 3.5% in non-smokers. Interestingly, our results confirmed that the negative effect of smoking still outweighed the established risk of steroids on healing.

This study set out to stratify the risk of medical-related complications as a separate outcome in long-term smokers. The pre-anaesthesia co-morbidity (ASA) score has been shown to be a reliable independent predictor of medical complications and mortality following surgery [23]. In our cohort of patients, most smokers were either ASA 3 or above when compared to non-smokers (64.4% vs 62.2%, *p* = 0.367). The higher proportion of ASA 3 and above in smokers is most probably related to their associated medical comorbidities because of long-term smoking, which include cardiopulmonary-related disease. Lifelong smokers have a 50% probability of developing COPD during their lifetime and studies have proven that COPD is an independent risk factor for pulmonary complications [24, 25]. Unsurprisingly, the proportion of patients suffering from COPD amongst smokers was also higher than non-smokers (14.3% vs 10.6%). Adjusting for patient comorbidities, the risk of specific medical-related complications was higher (*OR* = 1.15, 95% CI 0.97–1.36, *p* = 0.114) in long-term smokers but not of clinical significance. Furthermore, we also identified that the rate of pneumonia was higher in smokers (2.20% vs 1.50%, *p* = 0.085) and smokers had a 1.5-fold increase in primary pulmonary complications compared to non-smokers. Prolonged ventilation is a recognised risk factor for increased postoperative pulmonary complications [25]. As such, we identified a trend towards higher reintubation (and therefore prolonged ventilation) rate in smokers (1.5% versus 1.0%, *p* = 0.101) when compared to non-smokers but of not clinical significance. We believe that larger sample size would eventually confirm those trends.

Analysis of sepsis was conducted as a separate outcome due to its aetiology (either medical or surgical) being indistinguishable from the NSQIP database. This strategy was different to similar studies and provides a more precise outcome measure of complication profile in smokers. The likelihood of sepsis was 1.2-fold higher in smokers compared to non-smokers, but this was not clinically significant. We also know that patients returning to theatre, especially for AL, are most likely to present with symptoms of sepsis that is associated with longer length of hospital stay and trends towards a higher mortality rate [26]. The average LOS for those patients is approximately 75% greater than for most other conditions and dramatically increases with sepsis severity [27]. In our study, we identified a higher rate of return to operating room (4.8% vs 3.7%, *p* = 0.045), organ space infection (4.1% vs 3.1%, *p* = 0.034) and AL (3.5% vs 2.1%, *p* < 0.005) amongst smokers that also explains the increased likelihood of sepsis in this group of patients. AL is probably the most serious and feared complication specific to colorectal surgery as it is associated with significant morbidity and mortality [28, 29]. The literature reports a risk of AL post RH between 3 and 8% [30, 31]. From this cohort of selected patients, we found a lower overall rate of only 2.6%. This difference is likely attributed to our exclusion of emergency procedures and other unfavourable factors, such as preoperative sepsis and moribund patients not expected to survive without surgery (ASA 5 score), that could have had positively influenced outcome. Despite these exclusions, we identified that smokers had a 1.6-fold increased risk of AL compared to non-smokers. Sorensen and colleagues reported similar threefold increase in smokers after colorectal surgery, much higher due to the inclusion of left-sided colonic resections [32]. Once again, this outlines the importance of preoperative assessment and early smoking cessation to minimise this risk of AL.

As clinicians, we must acknowledge the increased risk of medical and surgical complications in smokers, as highlighted by this study. Preoperative risk stratification will enable health care professionals in minimising those risks, focussing on prevention rather than curative measures. Strategies that may be of benefit include preoperative smoking cessation programs that have demonstrated positive effects on post-surgical outcome, however not supported by others [33–36]. These inconsistencies likely reflect the differences in duration of preoperative smoking cessation, variability in types of surgery selected, and differing endpoints. For instance, the minimum duration of smoking cessation may vary between 2 and 8 weeks depending on the type of intended surgical procedure [37–40]. Additionally, extended timing between diagnosis and planned surgery may not always be possible, especially when dealing with colonic malignancies. Unsurprisingly, Sorensen and colleagues demonstrated that shorter-term smoking cessation failed to improve tissue and wound healing, as well as complication profile [33]. Extended smoking cessation schemes are therefore paramount for reducing the risk of post-operative complications and should remain an important target for quality and improvement [7]. The introduction of Enhanced Recovery After Surgery (ERAS) protocol specific to colorectal surgery has substantially improved post-operative outcomes in patients undergoing colorectal procedures [41]. Additional modifications of this ERAS programme for smokers may be considered, for example, with the introduction of a more aggressive approach to chest physiotherapy to minimise postoperative pulmonary complications and longer broad-spectrum antibiotics use, but also to minimise the risk of wound and septic complications. The potential success of these proposed strategies is probably best answered by observational or well-conducted randomised studies.

We acknowledge certain limitations in this study. For instance, definition of smoking as per the variable definition from the NSQIP database includes current smoking as a history of smoking within a year of surgery. This means that one who may have quit smoking several months before surgery would still be classified as a smoker. Secondly, there is continuous debate on the risk of AL based on the surgical technique (stapled versus handsewn) in the elective setting [42, 43]. This study does not account for the surgical technique nor surgeon experience that could have influenced the outcome of AL, or other complications. Thirdly, the use of preoperative antibiotics at induction is standard practice in gastrointestinal surgery. We assume that patients would have been administered the appropriate regimen as per local hospital protocol. However, this variable is unaccounted for in this study. The strength of our study is that 30-day outcomes were retrospectively reviewed from a large-scale prospectively maintained cohort of patients where all pre- and post-operative factors are objectively collected in a consistent and validated manner, by NSQIP hospital data coordinators. Additionally, we have used strict inclusion and exclusion criteria of the medical and surgical composite outcomes.

In conclusion, this study highlights important issues surrounding complications associated with smoking, post RH for bowel cancer. Smoking is an independent risk factor of wound complications, pulmonary complications, unplanned return to operating room, sepsis and AL. This study supports and supplements the current literature by providing a more in-depth analysis in the risk profile of long-term smokers, using composite outcomes that are clinically relevant to patients and clinicians. Surgeons who counsel patients undergoing RH should emphasize these increased risk of complications prior to elective surgery.
