# Correction to: Impact of preoperative smoking on patients undergoing right hemicolectomies for colon cancer

**DOI:** 10.1007/s00423-022-02528-2

**Published:** 2022-07-27

**Authors:** Sarit Badiani, Jason Diab, Evangelin Woodford, Pragadesh Natarajan, Christophe R. Berney

**Affiliations:** 1 Bankstown Lidcombe Hospital, Eldridge Rd, Bankstown, NSW 2200 Australia; 2 School of Medicine, University of New South Wales, Sydney, Australia; 3 School of Medicine, University of Notre Dame, Sydney, Australia; 4 Department of Surgery, Westmead Hospital, Westmead, NSW Australia

**Correction to: Langenbeck's Archives of Surgery.**

**https://doi.org/10.1007/s00423-022-02486-9**

The original version of this article unfortunately contained a small mistake in the body text which is “4416 44,160”.

The corrected number should be: “44160”.

The original article has been corrected.

